# Investigation and Optimization of Process Parameters on Growth Rate in Al_2_O_3_ Atomic Layer Deposition (ALD) Using Statistical Approach

**DOI:** 10.3390/ma18091918

**Published:** 2025-04-23

**Authors:** Dongqing Pan, Yu Lei

**Affiliations:** 1 Department of Engineering and Industrial Professions, University of North Alabama, Florence, AL 35632, USA; 2 Department of Chemical and Materials Engineering, University of Alabama in Huntsville, Huntsville, AL 35899, USA; yu.lei@uah.edu

**Keywords:** atomic layer deposition, Al_2_O_3_ thin film, growth rate, statistical approach, full factorial design of experiments

## Abstract

The improvement in ALD growth rate has always been challenging due to its slow atomic-scale depositions. Although Al_2_O_3_ ALD is one of the most widely used ALD processes, the effects of its process parameters on growth rate have not been systematically analyzed using statistical approaches. These statistical methods offer better efficiency and effectiveness compared to traditional techniques for studying complex processes like ALD. This paper presents a systematic investigation and optimization of four process parameters on growth rate of Al_2_O_3_ ALD thin films using a full factorial design of experiments (DOE) approach. Statistical analysis revealed that deposition temperature is the only statistically significant factor in Al_2_O_3_ ALD process, while argon gas flow rate, pulsing time and purging time are tested nonsignificant. Significant interactions were found between deposition temperature and purging time, and between pulsing time and purging time, with all other interactions being nonsignificant. Optimal process settings for higher deposition rate were identified: the temperature and gas flow rate are set at lower levels, while pulsing time and purging time are set at higher levels.

## 1. Introduction

Alumina thin film is studied as a model atomic layer deposition (ALD) process, owing to its high dielectric constant, high thermal stability and good adhesion on various wafer surfaces [1]. ALD has already been extensively adopted in silicon microelectronics and thin-film fabrications due to its highly self-limited depositions, perfectly uniform thin film surface geometry and accurately controlled film thickness [1]. Alumina thin film can be deposited by ALD using various precursors, including Trimethylaluminum (TMA), Dimethylaluminum isopropoxide (DMAI), Aluminum chloride (AlCl_3_) as Al sources, and water, ozone (O_3_) and H_2_O_2_ as oxygen sources [2]. The most studied precursors of alumina ALD are TMA and water, because of TMA’s high volatility and reactivity, allowing for efficient deposition across a wide temperature range [3,4].

Low throughput has always been a challenge for ALD applications due to its intrinsic nature of depositing materials at atomic level [5]. For instance, the typical growth rate for alumina ALD is around 1.1 Å/cycle depending on the deposition parameters [5,6,7,8]. Despite the concept of spatial ALD being adopted to improve the throughput and productivity of ALD process in recent years [7,9,10], considering traditional thermal ALD reactors are still extensively used in research and industrial settings, improving growth rate is still a pressing problem in the ALD community. The growth rate of ALD process is not only determined by the reactor designs, gas delivery systems, substrate placement, orientation and precursors [11,12], but also largely affected by process parameters, such as temperature, pulsing time, purging time, inert gas flow rate and chamber pressure [13,14].

Researchers have been exploring alumina ALD process by tuning those parameters to achieve higher growth rate. Groner et al. studied Al_2_O_3_ films deposited by ALD and the growth rate varied between 1.0 and 1.3 Å/cycle at temperatures from 33 to 177 °C using TMA and H_2_O [15]. Mousa et al. presented a study on the effects of temperature and gas flow rate on film growth of Al_2_O_3_ ALD using TMA and water, and the results showed higher growth rate (~1.5 Å/cycle) was observed when the gas flow rate was 5 slm, but the growth rate declined as the temperature was higher than 150 °C [16]. A study of the effect of deposition temperature and subsequent annealing time of ALD deposited Al_2_O_3_ films on silicon surface passivation using TMA and water showed that the film growth rate increased steadily from 0.8, 0.95 to 1.0 Å/cycle with the deposition temperature rises from 100, 200 to 300 °C [17].

Ylivaara et al. showed alumina ALD GPC increased from 0.73 to 0.94 Å/cycle when increasing ALD temperature from 110 to 250 °C, and after that it decreased to 0.90 Å/cycle when temperature increased to 300 °C [18]. Li and Ren presented their work on the effects of temperature and purging time of Al_2_O_3_ ALD process, and the growth rate slightly increased from 0.68 to 0.79 Å/cycle when deposition rate was increased from 100 to 150 °C. The purging time showed nonsignificant on growth rate as it increased from 3 to 9 s, the growth rate was kept at 0.78–0.79 Å/cycle [19]. Suyeon Kim et al. examined the growth rate and dielectric strength of Al_2_O_3_ ALD films at low temperatures (lower than 150 °C) and it showed that the growth rate of the Al_2_O_3_ films increased from 0.9 to 1.1 Å/cycle as temperature increased to 150 °C and then saturated beyond 150 °C [5]. Karnopp et al. studied the influence of TMA and water flow rate on the deposition rate of Al_2_O_3_ ALD at two different temperatures, and it showed that the deposition process reached saturation state as the precursor flow rate increased, and increasing the temperature to 200 °C slightly decreased the growth rate [20].

Review of the literature concludes that deposition temperature is the most studied factor in Al_2_O_3_ ALD, while other process factors such as pulsing time, purging time, inert gas flow rate and chamber pressure are much less investigated. Karnopp et al. studied the effect of the precursor flow rates on Al_2_O_3_ ALD deposition process [20] and Li and Ren included purge times in their study [19]. Despite deposition temperature being the most studied process parameter, no research was found to statistically confirm the significance of deposition temperature effect. Furthermore, none of the above work considered those process parameters all together systematically, and the interaction effects between the process factors were left uninvestigated.

Meanwhile, most of the reviewed publications adopted the one-factor-at-a-time (OFAT) method. In the OFAT method, a single process parameter, such as deposition temperature, is varied at different levels, sequentially, while other factors remain constant [21,22]. OFAT method requires a large number of deposition experiments in order to investigate all the process factors one by one, and this makes ALD experiments extremely time-consuming and costly.

Another major issue of OFAT method is the ignoring of interaction effects [22]. Interactions extensively exist in experiments when the effect of one parameter depends on the levels of another parameter [23]. For example, in Li and Ren’s work, the temperature effect on the growth rate was only investigated at a fixed level of purging time, and the interaction between the two factors was not studied [19]. Moreover, OFAT experiments are usually sequentially carried out, and hence, without randomization of the experiments order, the effect of extraneous parameters could be introduced into the response [22]. Lastly, in the published alumina ALD experiments, no work mentioned that replication was obtained, considering replication allows us to obtain an estimate of the experimental error and a more precise estimate of the effect of the parameters [22].

Statistical approaches can solve those problems by implementing carefully designed experiments and statistical analysis using design of experiments (DOE) methodologies. DOE applies statistics in all the experimental activities from planning, designing and implementing, to analyzing the experimental results [21]. Various DOE methods are available for different experimental goals, including full factorial designs, fractional factorial designs and response surface methodology (RSM). In a full factorial design, all possible combinations of factors and their levels are tested, so it offers comprehensive understanding of the main effects and all the interaction effects. However, full factorial designs become very costly and even impractical as the number of experiments grow exponentially with more factors. It is ideal for detailed and thorough analysis and optimization of a small number of factors (e.g., 2–4) [22].

Fractional factorial designs reduce the experimental costs and save experimental runs by testing a subset of combinations, but it loses information on some interactions. It is often used in screening experiments with many factors (e.g., 5–10) to identify the most influential ones, especially when experimental resources are limited. RSM is mainly used for optimization by modeling continuous factor effects with polynomial equations to find ideal response conditions when key factors have been already identified using methods like factorial designs. It often involves a series of designed experiments that fit a second-order polynomial to describe the relationship between the input factors and the response. It is a deal for fine tuning processes, optimizing product designs or determining the ideal conditions for achieving a target response [22,24]. To fully investigate a process such as ALD process, the best approach is to conduct a factorial DOE first to identify the significant effects, and then RSM can be used for further fine tuning of the processes [22].

DOE methods are not extensively adopted in ALD research, due to the lack of deeper statistical knowledge and confidence in allowing computer software to decide experiments [21]. Despite Al_2_O_3_ thermal ALD is one of the most studied ALD processes, very few papers used DOE to investigate its process parameters. Shendokar et al. used a two-level factorial design of experiments for the predictive evaluation of Al_2_O_3_ ALD process, in which two level of TMA pulsing time, water pulsing time, ozone pulsing time and 100–200 °C deposition temperature were investigated [25]. A research work presented by Dogan et al. used a two-level, eight-run fractional factorial method to identify the main parameters that affect the defect density of Al_2_O_3_ ALD thin film, and then the Bayesian optimization (BO) method was utilized to find the optimum values of the selected process parameters for rapid and adaptive optimization of the defect density [26].

As discussed earlier, a thorough understanding of the effects of ALD process parameters and their interactions using factorial designs is necessary before establishing optimal process parameters using methods such as RSM. As revealed by the literature review, little work has been done to study the main effects and interaction effects of Al_2_O_3_ ALD process. Therefore, in this work, a two-level (2^4^) full factorial DOE method is utilized to systematically investigate the main effects of four ALD process parameters (deposition temperature, inert gas flow rate, pulsing time and purging time) and their interactions on the growth rate of Al_2_O_3_ ALD thin films.

Despite two-level factorial DOE not capturing curvature effects or nonlinear effects, our primary goal in this study is to identify the significant factors and interactions. Considering multi-level factorial designs or response surface designs requiring significantly more experimental runs, a two-level factorial DOE can provide meaningful insights with desirable experimental efficiency [22,23]. With two replicates for each experimental run, 32 Al_2_O_3_ thin film samples are deposited using TMA and water as precursors by a commercial thermal ALD reactor. Statistical analysis is performed to identify the significant process parameters and their effects on growth rate. DOE analysis also reveals the potential interaction effects between the parameters, and response optimization is performed to find the optimal process condition for higher Al_2_O_3_ thin film growth rate. In addition, the work is expected to clear the obstacles of using statistical approaches in ALD experiments and lays out the factorial DOE fundamentals and analysis framework to promote the applications of statistical approaches in ALD community.

## 2. Statistical Approach and Full Factorial Design

### 2.1. Statistical Approach

Factors in ALD experiments are categorized based on their controllability. Controllable factors are those that researchers can directly control, such as deposition temperature, precursor pulse durations and purging times. On the other hand, uncontrollable factors are either too complex to control under normal operations, or of less research interest. Uncontrollable factors include variables such as batches of substrate, types of ALD reactors, environmental conditions (e.g., ambient temperature and humidity) and human-related influences such as different instrument operators.

The three common experimentation strategies are trial-and-error, OFAT and statistical approach. Typically, researchers using the trial-and-error method make educated guesses about the best combination of ALD process factors and their levels based on their understanding of the ALD process and experience. If the desired results are not achieved quickly, this process can become time-consuming and infeasible due to limited time, materials and personnels. The OFAT method, as previously discussed, has significant limitations, making it less effective and efficient to draw reliable conclusions from ALD experiments. Statistical DOE addresses these shortcomings by using statistical principles throughout the entire experimental process, providing more reliable and insightful conclusions [22,23].

The three fundamental principles of DOE are randomization, replication and blocking. Randomization aims to eliminate the influence of uncontrollable extraneous factors or errors by allocating raw materials and the sequence of ALD experimental runs randomly. Replication allows researchers to estimate experimental error by analyzing variations in replicated observations under identical experimental conditions [23], and improves the accuracy of factor estimates with averaged values [22]. Blocking is used to group homogeneous experimental runs into blocks, eliminating the influence of nuisance factors that are not a primary concern such as the batches of precursors and substrates in ALD experiments. By applying these principles, DOE minimizes experimental error and the effects from nuisance factors, and prevents misleading conclusions [23].

Factorial designs at two or three levels are commonly used in experimental designs [23], as these designs are highly efficient to study processes with multiple factors [22]. Furthermore, factorial designs allow for analyzing interaction effects, which are totally ignored in methods like OFAT; and because the effect of one factor is assessed across various levels of other factors, more valid conclusions across a range of experimental conditions can be obtained [22]. In this study, a factorial design is used to systematically explore all possible combinations of the four process parameter levels in Al_2_O_3_ ALD process and investigate the effects of both individual process parameters and their interactions on thin film growth rate.

### 2.2. Analysis of Variance (ANOVA)

The effect of the four targeted ALD process factors is termed as the main effect in DOE, which is defined as the variations in response (film growth rate) resulting from a change of that factor. The interaction effect between two factors is defined as the average difference in the effect of one factor across different levels of another factor. For example, the interaction effect between factors *A* and *B*, $I_{AB}$ is expressed as follows:

(1) $$\;I_{AB}=\frac{1}{2}\left(E_{A,B\left(+1\right)}-E_{A,B\left(-1\right)}\right)$$

where $E_{A,B(+1)}$ is the effect of factor *A* at high level of factor *B* and $E_{A,B(-1)}$ is the effect of factor *A* at low level of factor *B*.

Interaction effects can be analyzed using a graphical tool known as an interaction plot. In interaction plots, the response of one factor is plotted at different levels of another factor. If the lines in the interaction plot are parallel, no interaction exists between the two factors, meaning the effect of one factor does not rely on the level of the other factor. On the other hand, if the effect of one factor depends on the level of another, the lines are not parallel, indicating the presence of an interaction effect. The greater the departure from being parallel, the stronger the interaction between the factors [23].

To identify the factors that significantly impact the ALD growth rate, the most effective tool in the DOE is analysis of variance (ANOVA). The fundamentals of ANOVA are outlined as follows.

Consider a single factor experiment with *a* levels and *n* replicates. The observations can be represented using the following mean model:

(2) $$\;y_{ij}=\mu_{i}+\varepsilon_{ij}$$

where $i=1,2,\ldots ,a,\;j=1,2,\ldots ,n,\;y_{ij}\;$ is the *ij*th observation, $\mu_{i}$ is the mean of the *i*th factor level or treatment and $\;\varepsilon_{ij}$ is a random error item that incorporates all other sources of variability in the experiment including measurement, variability arising from uncontrolled factors, differences between the experimental materials, etc., and the general variabilities in the process (such as variability over time, effects of environmental variables, etc.).

The above mean model can be represented by an effect model by letting

(3) $$\;\mu_{i}=\mu +\tau_{i}$$

Hence, Equation (2) becomes the following:

(4) $$\;y_{ij}=\mu +\tau_{i}+\varepsilon_{ij}$$

where μ is the overall mean of the observations, and $\tau_{i}$ is *i*th treatment effect. This model is referred to as an effect model.

Note that the model random error $\varepsilon_{ij}$ is assumed to be normally and independently distributed with mean zero and variance ${\;\sigma }^{2}$ [22]. This means that the observations are following a normal distribution,

(5) $$\;y_{ij}\sim N(\mu +\tau_{i},\sigma^{2})$$

and the observations are mutually independent.

To determine whether the effect of the factor is significant on the response, that is, the treatment means $\mu_{i}$ are statistically different, the following null and alternative hypotheses are tested:

$$\;H_{0}:\mu_{1}=\mu_{2}=\ldots =\mu_{a}$$

(6) $$H_{A}:\mu_{i}\neq \mu_{j}\;for\;at\;least\;one\;pair\;\left(i,j\right)$$

To test the above hypotheses, consider the total sum of squares, which is defined as follows:

(7) $$\;SS_{T}=\sum_{i=1}^{a}\sum_{j=1}^{n}{\left(y_{ij}-\overset{-}{y_{..}}\right)}^{2}$$

where $\overset{-}{y_{..}}$ represents the grand average of all the observations, that is

(8) $$\;\overset{-}{y_{..}}=\frac{1}{an}\sum_{i=1}^{a}\sum_{j=1}^{n}y_{ij}=\frac{1}{N}\sum_{i=1}^{a}\sum_{j=1}^{n}y_{ij}=\frac{1}{N}y_{..}$$

and *N* = *an* is the total number of observations, and $y_{..}$ is the grand total of all the observations. $SS_{T}$ is used to measure overall variability in the observations, and note that it can be written as follows:

(9) $$\;\sum_{i=1}^{a}\sum_{j=1}^{n}{\left(y_{ij}-\overset{-}{y_{..}}\right)}^{2}=\sum_{i=1}^{a}\sum_{j=1}^{n}{\left[\left(\overset{-}{y_{i.}}-\overset{-}{y_{..}}\right)+\left(y_{ij}-\overset{-}{y_{i.}}\right)\right]}^{2}$$

where $\overset{-}{y_{i.}}$ is the average of the observations under the *i*th treatment, which is

(10) $$\;\overset{-}{y_{i.}}=\frac{1}{n}y_{i.}=\frac{1}{n}\sum_{j=1}^{n}y_{ij}$$

and $y_{i.}$ is the total of the observations under the *i*th treatment.

Expanding Equation (9) yields the following:

(11) $$\begin{array}{l} \;\sum_{i=1}^{a}\sum_{j=1}^{n}{\left(y_{ij}-\overset{-}{y_{..}}\right)}^{2}\\ \;\;\;=n\sum_{i=1}^{a}{\left(\overset{-}{y_{i.}}-\overset{-}{y_{..}}\right)}^{2}+\sum_{i=1}^{a}\sum_{j=1}^{n}{\left(y_{ij}-\overset{-}{y_{i.}}\right)}^{2}+2\sum_{i=1}^{a}\sum_{j=1}^{n}\left(\overset{-}{y_{i.}}-\overset{-}{y_{..}}\right)\left(y_{ij}-\overset{-}{y_{i.}}\right) \end{array}$$

Note that,

(12) $$\;\sum_{j=1}^{n}\left(y_{ij}-\overset{-}{y_{i.}}\right)=y_{i.}-\overset{-}{ny_{i.}}=0$$

Therefore, $SS_{T}$ becomes the following:

(13) $$\;\sum_{i=1}^{a}\sum_{j=1}^{n}{\left(y_{ij}-\overset{-}{y_{..}}\right)}^{2}=n\sum_{i=1}^{a}{\left(\overset{-}{y_{i.}}-\overset{-}{y_{..}}\right)}^{2}+\sum_{i=1}^{a}\sum_{j=1}^{n}{\left(y_{ij}-\overset{-}{y_{i.}}\right)}^{2}$$

Careful examination of the equation shows that the first item $n\sum_{i=1}^{a}{\left(\overset{-}{y_{i.}}-\overset{-}{y_{..}}\right)}^{2}$ is the sum of squares of the differences between the treatment averages and the grand average, denoted as $SS_{Treatments}$ (sum of squares due to treatments), and the second item, $\sum_{i=1}^{a}\sum_{j=1}^{n}{\left(y_{ij}-\overset{-}{y_{i.}}\right)}^{2}$ is the sum of squares of the differences of observations (replicates) within treatments from the treatment average, denoted as $SS_{E}$ (sum of squares due to error within treatments).

(14) $$\;SS_{T}=SS_{Treatments}+SS_{E}$$

Since there are *a* levels of the factor (and *a* treatment means), $SS_{Treatments}$ has *a* − 1 degrees of freedom and there are *n* replicates within each treatment, which provides *n* − 1 degrees of freedom to estimate the experimental error. With *a* treatments, there are *a*(*n* − 1) = *an* − *a* = *N* − *a* degrees of freedom for error.

Hence, the mean squares of treatments and errors are defined as follows:

(15) $$\;MS_{Treatments}=\frac{SS_{Treatments}}{a-1}$$

(16) $$MS_{E}=\frac{SS_{E}}{N-a}$$

Based on Cochran’s theorem, $MS_{Treatments}$ and $MS_{E}$ are independently distributed chi-square random variables. Therefore, if the null hypothesis of no difference in treatment means *H*_0_ is true, by the definition of *F* distribution, the ratio,

(17) $$\;F_{0}=\frac{MS_{Treatments}}{MS_{E}}=\frac{\frac{SS_{Treatments}}{a-1}}{\frac{SS_{E}}{N-a}}$$

follows the *F* distribution with *a* − 1 numerator degrees of freedom and *N* − *a* denominator degrees of freedom. Equation (17) is the test statistic for the hypothesis of no differences in treatment means.

Therefore, if

(18) $$\;F_{0}>F_{\alpha ,a-1,N-a}$$

where *α* is the level of significance, which is usually set as 0.05, the null hypothesis *H*_0_ will be rejected, which concludes that there are differences in the treatment means.

In practice, the *p*-value approach is used, and the *p*-value is the probability that the test statistic $F_{0}$ will take on a value that is at least as extreme as the observed value of the statistic when the null hypothesis *H*_0_ is true. If the *p*-value is larger than 0.05, the null hypothesis *H*_0_ is true, and the effect of a factor is concluded as not significant. Otherwise, the effect is statistically significant.

ANOVA is the same for experiments with more than one factor, and the ANOVA for a *k*-factor factorial design with $a_{i}$ levels for factor *i*, and *n* replicates for each treatment combination is summarized in Table 1.

However, before using ANOVA, it is necessary to check if the normality assumption is satisfied. In the effect model, the random error item $\varepsilon_{ij}$ is assumed to be normally and independently distributed. Validation of this normality assumption can be done by examination of residuals, which is defined as follows:

(19) $$\;e_{ij}=y_{ij}-{\hat{y}}_{ij}$$

where ${\hat{y}}_{ij}$ is a fitted value (obtained from a regression model) of the corresponding observation $y_{ij}$.

By plotting a histogram of the residuals, the normality assumption can be verified if this plot looks like a sample from a normal distribution centered at zero. However, due to the limited runs an ALD researcher can usually conduct, the histogram usually is not like normal distribution. A more precise method for verifying normality is the normal probability plot of residuals. Probability plot is a graphical tool used to evaluate whether sample data follows a hypothesized normal distribution by visually examining its linearity. The principle is that if the error term follows a normal distribution, then plotting the theoretical percentiles of the normal distribution against the observed sample percentiles of the residuals should yield an approximately linear trend [22].

To construct this plot, the residuals are first ranked from smallest to largest. Each residual, corresponding to the *i*th ranked observation *y*(*i*), is then plotted against its observed cumulative probability, calculated as *p*% = (*i* − 0.5)/*n ×* %, where *n* is the total number of residuals. Moderate departures from normality are not a major concern in ANOVA [22]. Other methods to assess the normality assumption include plots of the residuals against the fitted values and observation order, the residuals in the plots should be structureless and the plots should not reveal any obvious patterns [22].

In addition to ANOVA, the normal probability plot of effects can also be used to identify significant factors. The concept is straightforward: like random experimental errors, nonsignificant factors should follow a normal distribution as well. As a result, their effects will align along a straight line on the normal probability plot, whereas significant factors will depart from this line due to their nonzero means [22]. This method is particularly useful in single-replicate experiments where replicating each treatment is impractical or too costly. In such cases, ANOVA cannot be performed because with only one replicate for each treatment, it is impossible to estimate errors within treatments [22].

### 2.3. Full Factorial Design of Experiments

In this study, a 2^4^ full factorial DOE is employed to investigate the growth rate in the Al_2_O_3_ ALD process. The four factors are deposition temperature (A), inert gas flow rate (B), pulsing time (C) and purging time (D), each tested at two levels. With each run, two separate replicates of thin films are deposited. In this full factorial DOE, all the possible factor combinations are tested, resulting in a total of 16 experimental runs as shown in Table 2, in which the treatment combinations are arranged in standard order by introducing factors sequentially as (1), *a*, *b*, *ab*, *c*, *ac*, *bc*, *abc*, *d*, *ad*, *bd*, *abd*, *cd*, *acd*, *bcd* and *abcd*.

Table 2 is critical in evaluating the main effects. For example, the main effect of factor A is calculated by subtracting the average of the eight runs where *A* is at the low level ($\overset{-}{y_{A^{-}}}$), from the average of the eight runs where *A* is at the high level ($\overset{-}{y_{A^{+}}}$) as below,

(20) $$\begin{array}{l} \;A=\overset{-}{y_{A^{+}}}-\overset{-}{y_{A^{-}}}\\ =\frac{a+ab+ac+abc+ad+abd+acd+abcd}{8n}\\ \;\;\;\;-\frac{\left(1\right)+b+c+bc+d+bd+cd+bcd}{8n}\\ \;\;\;\;=\frac{contrast_{A}}{8n} \end{array}$$

where *n* is the number of replicates in each run, and contrast is defined as the difference between sums of the eight runs with *n* replicates when factor *A* is at high and low levels. The contrast is used to evaluate the sum of squares for the effect.

To estimate the interaction effects, sign of interaction effects must be constructed as shown in Table 3. Note that the signs of interaction effects are determined by multiplying the corresponding main effect signs. For instance, the signs of *AB* interaction are obtained by multiplying the signs of factors *A* and *B* across all 16 runs. Consequently, interaction effects can be estimated using the same approach for main effects. For example, the effect of *AB* interaction is calculated as below,

(21) $$\begin{array}{l} \;AB=\overset{-}{y_{AB^{+}}}-\overset{-}{y_{AB^{-}}}\\ =\frac{\left(1\right)+ab+c+abc+d+abd+cd+abcd}{8n}\\ -\frac{a+b+ac+bc+ad+bd+acd+bcd}{8n}\\ \;\;\;\;\;\;=\frac{contrast_{AB}}{8n} \end{array}$$

where contrast of *AB* is the difference between sums of the eight runs with *n* replicates when interaction *AB* is at high and low levels.

Using the contrasts of effects, the sums of squares for the effects can be calculated as,

(22) $$\;SS=\frac{(contrast{)}^{2}}{16n}$$

With two levels for each factor, the main effects and interaction effects have only one degree of freedom. Therefore, the mean square of two-level experiments can be calculated as,

(23) $$\;MS=\frac{SS}{DF}=SS$$

and the test statistic is calculated as,

(24) $$\;F_{0}=\frac{MS_{Effect}}{MS_{Error}}=\frac{SS_{Effect}}{SS_{Error}}$$

In DOE, regression models are used to express the results of the experiment. Using two-factor experiments as an example, a regression model can be constructed as below,

(25) $$\;y=\beta_{0}+\beta_{1}x_{1}+\beta_{2}x_{2}+\beta_{12}x_{1}x_{2}+\varepsilon$$

where *x*_1_ is the first factor (*A*), *x*_2_ is the second factor (*B*), *x*_1_*x*_2_ is the interaction between the two factors, the *β*’s are regression coefficients and *ε* is a random error term.

To fit the regression mode, the intercept can be calculated as the grand average of all the observations, and the regression coefficients are one-half the corresponding main effect or interaction effect estimates, because the effect estimate is based on a two-unit change (from −1 to +1). With the regression model, the fitted value can be estimated, and the difference between the observations and fitted values are residuals.

To validate the regression model, ANOVA can be conducted on the model. The total sum of squares for the full model is determined by adding sums of squares of all main effects and interactions. For a two-factor regression model, the sum of squares for the full model can be calculated as below,

(26) $$\;SS_{Model}=SS_{A}+SS_{B}+SS_{AB}$$

Therefore, the test statistic for the model can be evaluated as,

(27) $$\;F_{0}=\frac{MS_{Model}}{MS_{E}}$$

The degree of freedom for the regression model is the sum of the degree of freedom from all main and interaction effects. The *F* statistic for a two-factor factorial design is testing the hypotheses as below:

$$\;H_{0}:\beta_{1}=\beta_{2}=\beta_{12}=0$$

(28) $$H_{A}:at\;least\;one\;\beta \neq 0$$

The ordinary *R*^2^ statistic can be used to evaluate the regression model, and it measures the proportion of total variability explained by the model as,

(29) $$\;R^{2}=\frac{SS_{Model}}{SS_{Total}}$$

Because the ordinary *R*^2^ value always increases as more factors or interactions are added to the model, even if they are not statistically significant, adjusted *R*^2^ is introduced,

(30) $$\;{R_{Adj}}^{2}=1-\frac{SS_{E}/DF_{E}}{SS_{Total}/DF_{Total}}$$

where $DF_{E}$ and $DF_{Total}$ are the degrees of freedom for the error and total, respectively. The adjusted *R*^2^ value is more accurate because it accounts for the size of the model, and it decreases if more nonsignificant effects are included in the model.

Another statistic is the prediction error sum of squares (PRESS), which evaluates how well the model predicts new data. To quantify the model prediction accuracy, the prediction *R*^2^ is calculated using the following equation,

(31) $$\;{R_{\mathrm{Pr}ed}}^{2}=1-\frac{PRESS}{SS_{Total}}$$

Prediction *R*^2^ represents the portion of the variability in new data that the regression model can explain [22].

## 3. Experimental Details

In this study, Al_2_O_3_ thin films are deposited on silicon (100) substrates with sizes approximately 10 mm × 10 mm. The aluminum precursor is 98% TMA from Strem Chemicals, Inc. (Newburyport, MA, USA), while 99.999% water (also from Strem Chemicals, Inc.) serves as an oxidizer. The substrate preparation process involves the following steps. The substrates are first immersed in acetone for 10 min to remove organic residues from the surface, then rinsed with methanol to eliminate any remaining acetone, followed by a final rinse with deionized water to ensure the removal of all residual solvents, and finally, they are dried using nitrogen gas before being placed in the ALD reactor for deposition.

**Figure 1:**
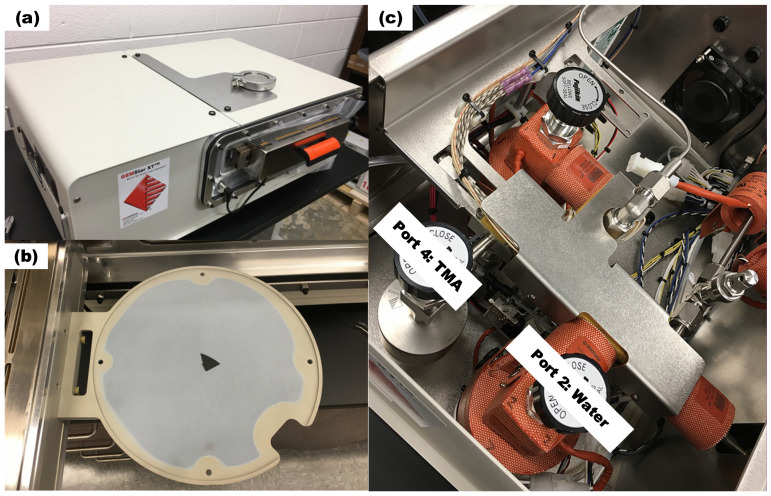
(**a**) Arradiance GEMStar XT Thermal ALD reactor (**b**) wafer holder and (**c**) precursor bottle installation and ALD port assignment to TMA and water.

The experiments are conducted by the Arradiance GEMStar XT Thermal ALD reactor (Arradiance, Littleton, MA, USA) as shown in Figure 1a, which is equipped with four precursor bottle ports, with water assigned to Port 2 and TMA to Port 4 as shown in Figure 1c. The reactor can accommodate wafers up to 200 mm in diameter as shown in Figure 1b and operate at temperatures up to 300 °C. Precursors are introduced into the chamber through inlet holes and exit through outlet pipe, which is connected to a vacuum pump. To ensure adequate precursor delivery, both the TMA (Port 4) and water (Port 2) bottles are heated to 30 °C. During experiments, the chamber pressure is maintained around 100 mTorr.

Argon (Ar) is used as the purging gas, with its flow rate regulated by a mass flow controller. In this study, Ar flow rates of 10 and 20 sccm are tested as controlling factors. The chamber temperature is maintained at either 175 or 275 °C, while the pulsing times for TMA and water are set the same, at 20 and 40 ms, based on the manufacturer’s recommendation for matched precursor injections. Additionally, the purging time is controlled between 10 and 20 s as another controlling factor in the experiments.

The levels of the controlling factors are summarized in Table 4. The levels of the factors were selected around the manufacturer’s recommended values (175 °C for deposition temperature, 10 sccm for Ar flow rate, 20 ms for both TMA and water pulsing times and 10 s for purging time). To be specific, in this study the recommended settings are set as low level of the factors, which serve as a well-established baseline to ensure the process operates within known and stable conditions. A higher level of the four factors (275 °C for deposition temperature, 20 sccm for Ar flow rate, 40 ms for both TMA and water pulsing time and 20 s for purging time) is selected to test the main and interaction effects of the four factors on deposition rate.

Silicon substrates are placed at the center of the wafer holder as shown in Figure 1b and two separate substrates are deposited for each experimental setting for 200 cycles, and the deposited film thickness is measured using an Alpha-SE spectroscopic ellipsometer (J. A. Woollam, Lincoln, NE, USA).

## 4. Results and Discussions

### 4.1. Experiment Results

In this research, 32 experimental runs are conducted following the 24 full factorial DOE. The experiment runs and results with measured GPC, fitted GPC and residuals are presented in Table 5. All the 32 experimental runs are randomized by MiniTab (version 20.3) as presented in Table 5. Precursors and silicon substrates are from the same batch, so there is only one block in the experiments.

**Figure 2:**
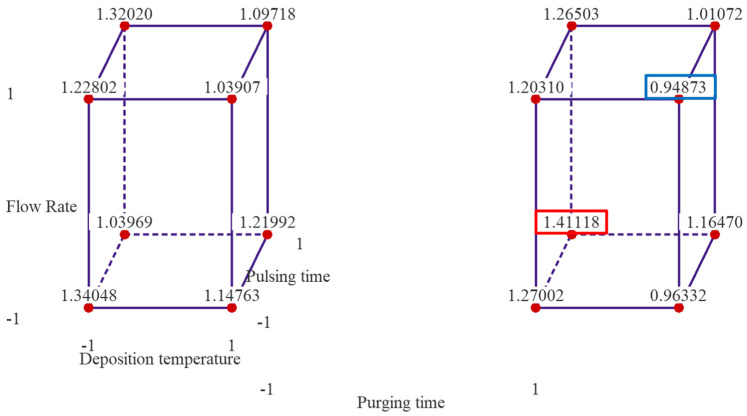
Cube plots of the experimental results with mean GPC values of the 16 treatment combinations. Note: the left cube is when purging time is at low level and right one is when purging time is at high level.

Figure 2 presents cube plots with the mean GPC values of two replicates for the 16 treatment combinations. The three axes of the cubes are temperature, flow rate and pulsing time, and the left cube is when purging time is at low level, and right one is when purging time is at high level. The cube plots reveal that the highest GPC is ~1.411 Å/cycle observed when the deposition temperature and flow rate are set at low levels, and pulsing time and purging time are set at high levels. The lowest average thickness of alumina thin film is ~0.949 Å/cycle when the temperature, flow rate and purging time are set at high level, and pulsing time is set at low level.

### 4.2. Regression Model

By performing a factorial regression, the residuals can be calculated and the normality assumption of the random error in the effect model can be verified. The regression model of the data is shown below,
GPC(Å/cycle) =1.1668 −0.0929 Deposition Temperature −0.0278 Flow Rate +0.0243 Pulsing Time −0.0122 Purging Time −0.0222 Deposition Temperature × Flow Rate +0.0250 Deposition Temperature × Pulsing Time −0.0398 Deposition Temperature × Purging Time +0.0100 Flow Rate × Pulsing Time −0.0199 Flow Rate × Purging Time +0.0340 Pulsing Time × Purging Time

Note that all the factors and up to two-way interactions are included in the model, as three-way or higher interactions are not practical or important in real-life settings [23]. The response is Al_2_O_3_ film growth rate, and the coefficient of each effect is estimated as half of the effect as shown in Table 6. The *R*^2^ value is 78.64%, which is acceptable considering the goal of the experiments is to identify the significant factors [22]. The relatively low *R*^2^ value is caused by missing three-way or higher interactions in the regression model.

**Figure 3:**
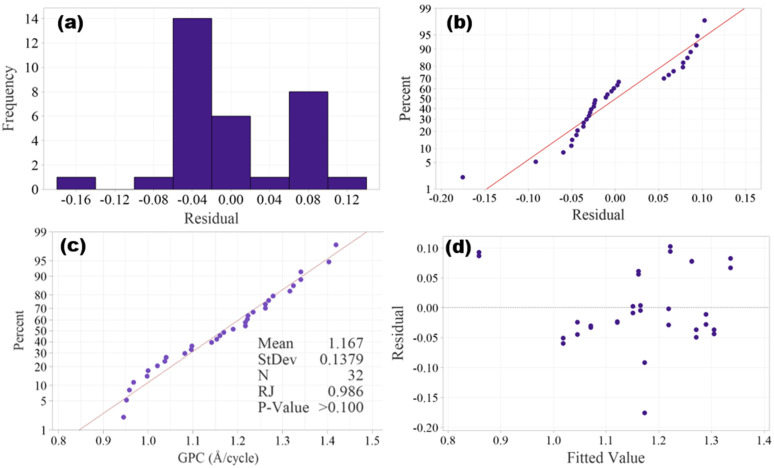
Plots of residuals: (**a**) histogram of residuals, (**b**) normal probability plot of residuals, (**c**) Ryan–Joiner test probability plot and (**d**) plot of residuals vs fitted values.

Before performing ANOVA, the assumptions of normality and homoscedasticity must be verified. In DOE, the assumption of normality can be verified by either graphical methods, such as histogram of residuals and normal probability plot, or statistical tests, such as Ryan–Joiner test, Shapiro–Wilk test, Kolmogorov–Smirnov test and Anderson–Darling test. Histogram of residuals is shown in Figure 3a, and it is hard to tell if a normal distribution is resembled due to the small size of the data. The normal probability plot as shown in Figure 3b shows the residuals roughly follow along a line, confirming that the normality assumption is satisfied.

Like the Shapiro–Wilk test, the Ryan–Joiner test is suitable for small sample sizes (*n* ≤ 50), and it is performed to further validate the normality. The Ryan–Joiner test probability plot is shown in Figure 3c, in which the data points closely follow the straight reference line, which implies that the experimental data is normally distributed. The Ryan–Joiner coefficient (RJ) is 0.986, which is very close to 1, and the *p*-value is >0.05 (fail to reject normality), both further validating the normality assumption.

The assumption of homogeneity of variances (homoscedasticity) assumes that the spread of residuals should be constant across all factor levels. Graphical methods such as residuals vs. fitted values plot and Levene’s test plot can be used to verify the homogeneity of variances. The plot of residuals vs. fitted values in Figure 3d shows that the spread of residuals appears random without any patterns, and hence the homoscedasticity is validated.

### 4.3. Significance of Factors and Interactions

With the normality and homoscedasticity assumptions verified, the ANOVA of the regression model is performed to determine the significance of the main effects and up to two-way interaction effects. The ANOVA results are summarized in Table 7. Each factor only has two levels, and hence the degree of freedom (DF in Table 7) of all the effects is one, and the four main effects form the linear portion of the model and six two-way interactions resemble the nonlinear portion of the model. Therefore, the model has 10 DFs. With a total of 32 runs in the experiments, there are 31 DFs. Therefore, the error has 21 DFs. Note that mean squares are the sum of squares divided by DF, and *F*-Value is evaluated by Equation (24).

ANOVA shows that the effects of deposition temperature, and two interactions between deposition temperature and purging time, pulsing time and purging time have *p*-values less than 0.05, and therefore, they are concluded as significant factors. Flow rate, pulsing time, purging time and other interactions are tested nonsignificant in this study.

**Figure 4:**
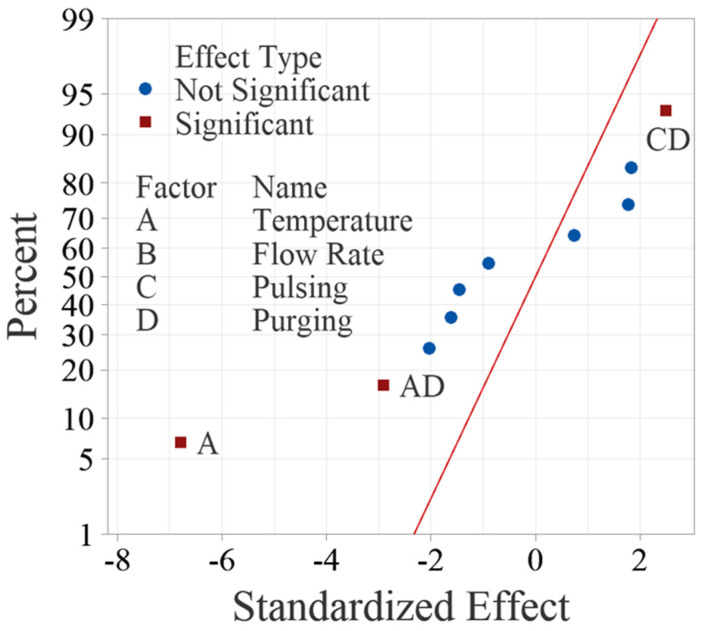
Normal probability plot of standardized effects for the main factors and up to two-way interactions.

The normal probability plot of these effects as shown in Figure 4 can also be used to confirm the significance of the effects. Figure 4 shows the normal plot of the standardized effects, which are calculated by dividing the effects using the standard deviation of the data. The standardized effects allow for comparison between different factors even if their units are dissimilar. Like random effects, nonsignificant main and interaction effects tend to fall along a straight line, while significant effects fall off the straight line. The normal probability plot of the effects yields the same results from ANOVA, confirming that factor A, interactions AD and CD are significant.

**Figure 5:**
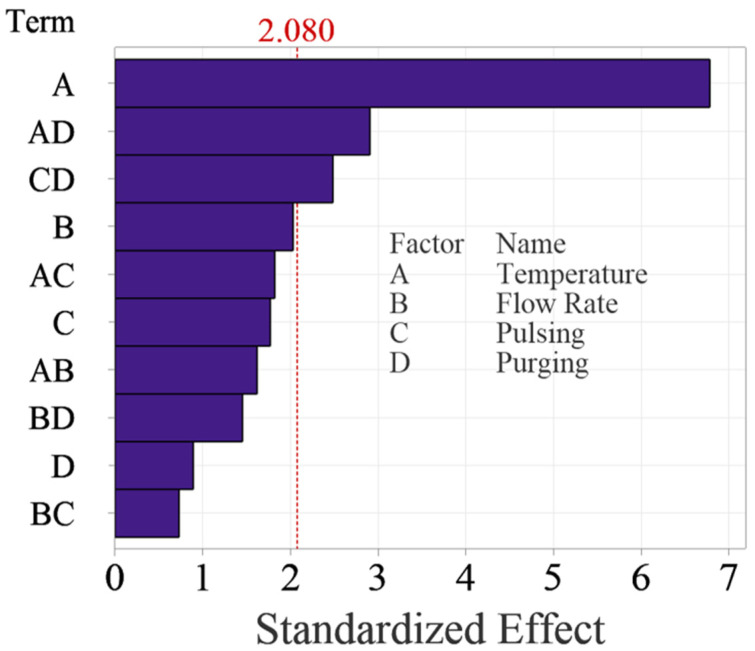
Pareto Chart of standardized effects for the main factors and up to two-way interactions.

Figure 5 shows the Pareto Chart of the standardized effects, which is more intuitive to examine the significance of the effects. The Pareto chart shows the absolute values of the standardized effects from the largest effect to the smallest effect with a reference line to indicate which effects are statistically significant. The reference line is determined based on the selected significance level, typically 0.05 for a 95% confidence level. Any effect that extends past this reference line is potentially significant. The Pareto Chart reaches the same conclusions about the significance of the effects. It is worth noting that temperature is the most significant factor, which surpassed the reference significantly, while the two interactions are relatively weaker, as they are close to the reference line.

### 4.4. Main Effects and Interaction Effects

**Figure 6:**
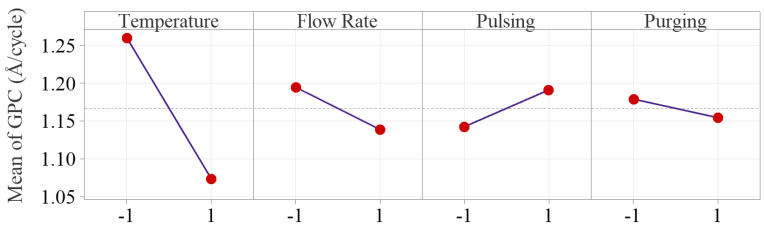
Main effect plots of the four process parameters. The inert gas flow rate, pulsing time and purging time have much less effects on GPC compared to deposition temperature.

**Figure 7:**
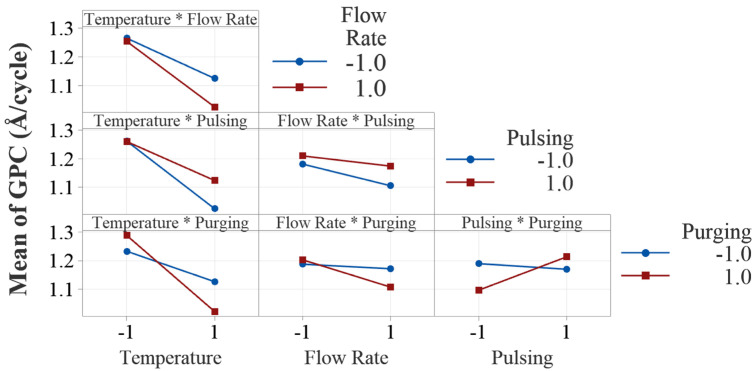
Interaction plots of the four process parameters. All the interaction plots are not parallel, indicating evident interaction effects.

To examine how the factors influence the Al_2_O_3_ thin film deposition rate, the main and interaction effect plots are constructed as shown in Figure 6 and Figure 7, respectively. The main effect plots indicate that the inert gas flow rate, pulsing time and purging time have much less effect on GPC compared to deposition temperature.

Deposition temperature is tested as the only significant main factor in Al_2_O_3_ ALD process, and the deposition temperature effect plot shows that Al_2_O_3_ growth rate drops significantly from 1.26 to 1.07 Å/cycle, as temperature increases from 175 to 275 °C. The result matches with the research work in the literature [15,16,17,20]. The decreasing growth rate is primarily due to the reduction of OH groups on the substrate surface. As temperature increases, the temperature-dependent desorption of water from the substrate surface is enhanced, particularly at lower temperatures. This reduction in surface OH groups limits the available reactive sites for precursor adsorption, and therefore a decrease of the overall deposition rate is observed [27,28].

Flow rate effect on growth rate is tested not statistically significant, but the effect plots show that increasing Ar gas flow rate from 10 to 50 sccm slightly decreases the average growth rate from 1.19 to 1.14 Å/cycle. Inert gas in ALD process serves two purposes: carrying precursors into the reactor chamber (positively affecting growth rate) and purging the precursor residuals out of the system (negatively influencing the surface reactions). The overall effect of Ar gas flow rate on the deposition process is balanced by the two effects, which makes the overall effect of inert gas flow rate nonsignificant.

Pulsing time is also concluded as a nonsignificant factor in our study. The effect plot indicates longer pulsing time slightly increases the average growth rate from 1.14 to 1.19 Å/cycle. This is attributed to the extra precursors injected into the reactor with longer pulsing time. Increasing pulsing time allows for more precursors to enter the reactor chamber, providing more reactants for surface reactions, and subsequently enhancing the deposition rate until saturation is reached [28,29,30]. Therefore, the effect of pulsing time relies on the fact whether the growth is saturated with sufficient precursors being injected into the chamber [28,30]. In our study, the relatively weak effect of pulsing time can be attributed to the fact that the selected levels are centered around the manufacturer’s recommended saturation pulsing time (20 ms for TMA and water). Therefore, increasing pulsing time beyond the saturation time can only result in a slight increase in growth rate.

The effect of purging time is also very weak, and the average GPC only decreases from 1.18 to 1.15 Å/cycle as the purging time increases from 10 to 20 s. The result matches with the work done by Li and Ren in which it showed the purging time is a nonsignificant factor on growth rate as the purge time increased from 3 to 9 s, and the growth rate was between 0.78–0.79 Å/cycle [19]. The decrease in deposition rate is primarily attributed to precursor residuals being more effectively removed from the chamber, which reduces gas-phase chemical vapor deposition (CVD)-type depositions [31]. Since CVD reactions compromise the surface quality of thin film in the ALD process, the reduction in deposition rate at a longer purging time is compensated by minimizing unwanted CVD depositions.

One of the advantages of DOE is its ability to assess interaction effects in the experiments. Figure 7 presents the two-way interaction effect plots in this study. Visual examination of parallelism of the two lines in the interaction plots indicates the existence of potential interaction effects between all the process factors. However, ANOVA shows that only two interactions, temperature × purging time and pulsing time × purging time, are statistically significant.

The interaction between temperature and purging time shows that the effect of deposition temperature on the deposition rate depends on the level of purging time. The decreasing effect of deposition temperature on growth rate is more pronounced when the purging time is at a high level (20 s). This can be attributed to the fact that with longer purging time, the desorpted water molecules at higher temperatures are more effectively cleaned out, resulting in lower growth rates because of excessive loss of the OH groups from the surface. At higher temperatures, volatile byproducts may desorb more quickly and potentially reduces the required purging time. At lower temperatures, byproducts may desorb more slowly, needing longer purging time to prevent contamination or unwanted reactions in subsequent cycles.

The interaction between pulsing time and purging time is also tested statistically significant by ANOVA. It is discussed that the effect of pulsing time on growth rate is positive. However, it is interesting to note that when purging time is shorter, increasing pulsing time decreases the growth rate slightly as shown in the interaction plot. Similarly, when pulsing time is shorter (20 ms), increasing purging time decreases the growth rate, but at higher level of pulsing time, it increases the growth rate. If purging is insufficient, residual precursor may cause unintended chemical reactions, contamination or non-ideal growth. On the other hand, excessive purging can reduce precursor adsorption efficiency, and results in an incomplete monolayer and lower growth rate. The interaction effects give us more insights into the effect of a factor when it is influenced by other factors in ALD processes.

### 4.5. Process Parameters Optimization

To find the optimal process parameter settings for growth rate, a reduced regression model is constructed by removing the nonsignificant effects. Since interactions between temperature, pulsing time and purging time are significant, these three factors, together with interaction between temperature and purging time and interaction between pulsing time and purging time are included in the reduced regression model shown below,
GPC (Å/cycle)=1.1668 −0.0929 Deposition temperature +0.0243 Pulsing time −0.0122 Purging time −0.0398 Deposition temperature × Purging time + 0.0340 Pulsing time × Purging time

**Figure 8:**
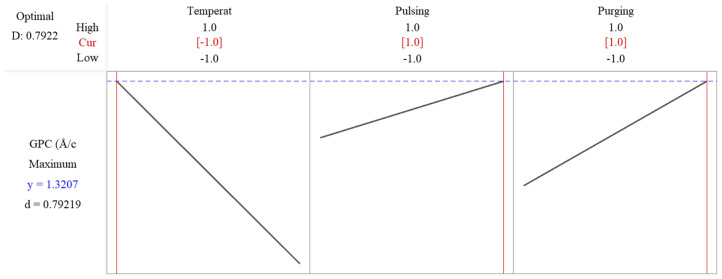
Response optimization shows the predicated maximum growth rate is 1.3207 Å/cycle when deposition temperature is set at low level, while pulsing time and purging time are set as high level.

The response optimization method in Minitab is used to identify the optimal settings of input factors that yield the best possible response in this factorial design. The method applies a desirability function, where each response is assigned a desirability value (D, ranging from zero to one), based on how well it meets the goal. The best combination is identified by maximizing the overall desirability score. The desirability value is shown as 0.7922 in Figure 8, which means that the response is achieving 79.22% of the ideal target. The predicated maximum growth rate is 1.3207 Å/cycle as shown in Figure 8, when deposition temperature is set at low level, while pulsing time and purging time are set as high level. Since flow rate is not significant, it is excluded in the reduced model, and to save resources, it can be set as low level, 10 sccm.

**Figure 9:**
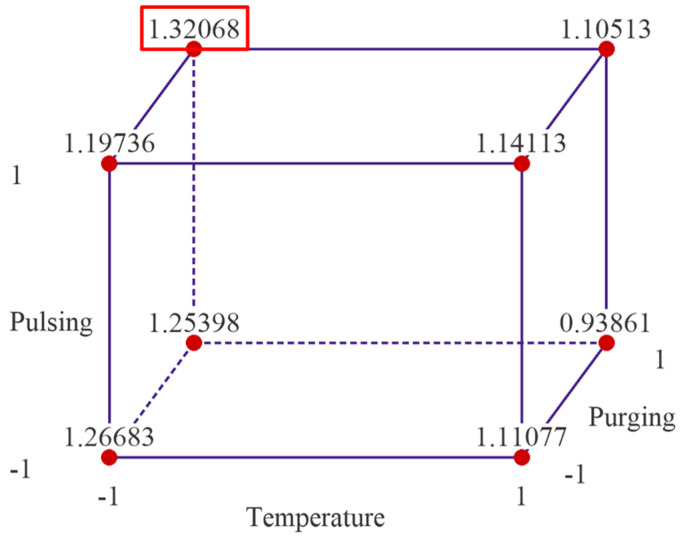
Cubic plot of the fitted data from the reduced regression model.

The cube plot of fitted means from the reduced regression model is shown in Figure 9. The cube plot yields the same result as the experimental data presented in Figure 2, indicating the optimal parameter setting is when temperature is set at low level, 175 °C, pulsing time is set at high level 40 ms for both TMA and water, and purging time is set at high level, 20 s.

**Figure 10:**
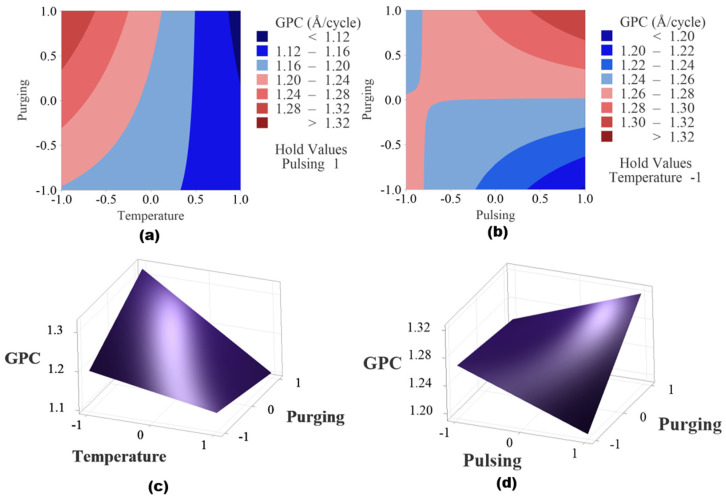
(**a**) Contour plot of temperature and purging time when pulse time is set as high level, (**b**) contour plot of pulsing time and purging time when temperature is held at low level, (**c**) surface plot of temperature and purging time when pulse time is set as high level and (**d**) plot of pulsing time and purging time when temperature is held at low level.

Figure 10 shows the contour plots and surface plots of the two significant interaction effects. When pulsing time is held at a high level as shown in Figure 10a,c, the higher GPC region is observed when deposition temperature is at low level and purging time is at high level. When temperature is fixed at a lower level, the higher GPC region is found when purging time and pulsing time are both at high level shown in Figure 10b,d.

It is noted that the effects of those factors and interactions on Al_2_O_3_ ALD growth could be nonlinear and complex. For example, previous work on Al_2_O_3_ ALD thin films using TMA and water showed that lowering substrates temperature too much, i.e., less than 150 °C, decreases the deposition rate significantly as there is not enough energy to initiate the surface reactions [1,6]. The effect of temperature on growth rate is often nonlinear due to competing effects; increasing temperature can enhance surface reactions and precursor decomposition, but excessive heat may lead to precursor desorption or decomposition before surface reactions, which thus results in reduction of GPC.

Increasing TMA and water pulsing time does not always increase the deposition rate. As discussed earlier, when the substrate surface receives sufficient precursors, the surface reaction will be saturated and the deposition rate will remain constant. A two-level design, with only a low and a high setting for each factor, is not capable of identifying intermediate optimal or nonlinear trends unless center points or higher-order terms are included. To address this limitation, future research can be done by extending the design to a central composite design (CCD) or response surface methodology (RSM) design, which introduces center points and allows modeling of quadratic terms, so that a more accurate exploration of potential nonlinearities can be done and a precise optimization of process parameters like temperature in ALD systems can be conducted. Nonetheless, the two-level factorial DOE offers invaluable information about the significance of the factors and their interactions for further investigations.

To further optimize Al_2_O_3_ ALD process for higher process efficiency, alternative precursor choices and novel reactor designs can also be explored. Selecting precursors with higher volatility, improved ligand structures and reduced decomposition byproducts can minimize contamination and improve growth kinetics. Novel reactor designs, such as spatial ALD for high-throughput processing, plasma-enhanced ALD for lower-temperature deposition and batch or roll-to-roll systems for scalability, offer improved deposition control.

## 5. Conclusions

By implementing a 2^4^ full factorial DOE, this study presented a systematic investigation of the effects of four ALD process parameters (deposition temperature, inert gas flow rate, pulsing time and purging time) on the growth rate of Al_2_O_3_ thin films fabricated by thermal ALD process using TMA and water as precursors. A comprehensive statistical analysis was conducted to evaluate the significance of each process factor and their interactions. A regression model with up to two-way interactions was developed, and the plots of residuals confirmed the normality assumption in the regression model.

Both ANOVA and Pareto chart revealed that deposition temperature is the only significant factor at a 95% confidence level, while inert gas (argon) flow rate, pulsing time and purging time are statistically nonsignificant in Al_2_O_3_ ALD process. Interaction analysis showed that, despite all of the interactions that existed between those process factors, only two interactions (temperature × purging time and pulsing time × purging time) are statistically significant. The analysis of deposition temperature effect indicated that increasing deposition temperature reduces the growth rate because of the enhanced desorption of OH groups from the substrate. Increasing inert gas flow rate slightly decreases the growth rate because of the enhanced purging effect of precursor residuals. Longer purging time decreases the growth rate by removing more precursor residuals, resulting in less CVD-type depositions. Increasing pulsing time leads to a higher growth rate because it introduces more precursors into the reactor chamber. The observed significant interactions, temperature × purging time and pulsing time × purging time show that the effects of deposition temperature and pulsing time on the growth rate depend on the level of purging time.

Optimal process conditions were also identified using response optimization method. Higher growth rate is achieved when the temperature and flow rate are set at low level, while pulsing time and purging time are kept at high levels. It is important to note that despite the effects of these parameters may not be strictly linear, and further experiments exploring additional factor levels are necessary for a more comprehensive understanding of those effects in Al_2_O_3_ ALD process, this full factorial study and its statistical analysis provided valuable insights into the significance of individual factors and their interactions.

## Figures and Tables

**Table 1:** The ANOVA table for a *k*-factor factorial design with $a_{i}$ levels for factor *i*, and *n* replicates for each treatment combination.

Source of Variations	SS	DF	MS	*F*_0_
Factor i (i = 1, 2…, *k*)	$SS_{i}\;$	$a_{i}$ − 1	$MS_{i}=\frac{SS_{i}}{a_{i}-1}\;$	$F_{0}=\frac{MS_{i}}{MS_{E}}$
Interaction ij (i, j = 1, 2…, *k*, and i≠j)	$SS_{ij}$	($a_{i}$ − 1)($a_{j}$ − 1)	$MS_{ij}=\frac{SS_{ij}}{\left(a_{i}-1)(a_{j}-1\right)}\;$	$F_{0}=\frac{MS_{ij}}{MS_{E}}$
Error	$SS_{E}\;$	${a_{1}a_{2}\ldots a}_{k}$(*n* − 1)	$MS_{E}=\frac{SS_{E}}{{a_{1}a_{2}\ldots a}_{k}\left(n-1\right)}$	
Total	$SS_{T}\;$	${a_{1}a_{2}\ldots a}_{k}\;$*n* − 1		

**Table 2:** Four-factor, two-level full factorial experiments notations following the standard runs.

Run	*A*	*B*	*C*	*D*	Run Label
1	−1	−1	−1	−1	*(1)*
2	+1	−1	−1	−1	*a*
3	−1	+1	−1	−1	*b*
4	+1	+1	−1	−1	*ab*
5	−1	−1	+1	−1	*c*
6	+1	−1	+1	−1	*ac*
7	−1	+1	+1	−1	*bc*
8	+1	+1	+1	−1	*abc*
9	−1	−1	−1	+1	*d*
10	+1	−1	−1	+1	*ad*
11	−1	+1	−1	+1	*bd*
12	+1	+1	−1	+1	*abd*
13	−1	−1	+1	+1	*cd*
14	+1	−1	+1	+1	*acd*
15	−1	+1	+1	+1	*bcd*
16	+1	+1	+1	+1	*abcd*

**Table 3:** Sign of interaction effects in 2^4^ full factorial DOE.

Labels	Run	*A*	*B*	*AB*	*C*	*AC*	*BC*	*ABC*	*D*	*AD*	*BD*	*ABD*	*CD*	*ACD*	*BCD*	*ABCD*
*(1)*	1	−1	−1	+1	−1	+1	+1	−1	−1	+1	+1	−1	+1	−1	−1	+1
*a*	2	+1	−1	−1	−1	−1	+1	+1	−1	−1	+1	+1	+1	+1	−1	−1
*b*	3	−1	+1	−1	−1	+1	−1	+1	−1	+1	−1	+1	+1	−1	+1	−1
*ab*	4	+1	+1	+1	−1	−1	−1	−1	−1	−1	−1	−1	+1	+1	+1	+1
*c*	5	−1	−1	+1	+1	−1	−1	+1	−1	+1	+1	−1	−1	+1	+1	−1
*ac*	6	+1	−1	−1	+1	+1	−1	−1	−1	−1	+1	+1	−1	−1	+1	+1
*bc*	7	−1	+1	−1	+1	−1	+1	−1	−1	+1	−1	+1	−1	+1	−1	+1
*abc*	8	+1	+1	+1	+1	+1	+1	+1	−1	−1	−1	−1	−1	−1	−1	−1
*d*	9	−1	−1	+1	−1	+1	+1	−1	+1	−1	−1	+1	−1	+1	+1	−1
*ad*	10	+1	−1	−1	−1	−1	+1	+1	+1	+1	−1	−1	−1	−1	+1	+1
*bd*	11	−1	+1	−1	−1	+1	−1	+1	+1	−1	+1	−1	−1	+1	−1	+1
*abd*	12	+1	+1	+1	−1	−1	−1	−1	+1	+1	+1	+1	−1	−1	−1	−1
*cd*	13	−1	−1	+1	+1	−1	−1	+1	+1	−1	−1	+1	+1	−1	−1	+1
*acd*	14	+1	−1	−1	+1	+1	−1	−1	+1	+1	−1	−1	+1	+1	−1	−1
*bcd*	15	−1	+1	−1	+1	−1	+1	−1	+1	−1	+1	−1	+1	−1	+1	−1
*abcd*	16	+1	+1	+1	+1	+1	+1	+1	+1	+1	+1	+1	+1	+1	+1	+1

**Table 4:** Four factors of Al_2_O_3_ ALD with high- and low-level values in the 2^4^ full factorial DOE.

Factor Label	Factor Name (Unit)	Low Level Value (−1)	High Level Value (1)
*A*	Temperature (°C)	175	275
*B*	Inert gas flow rate (sccm)	10	20
*C*	Pulse time (ms)	20	40
*D*	Purge time (s)	10	20

**Table 5:** Experiment runs and results with measured GPC, fitted GPC, and residuals.

Label	Standard Order	Run Order	Blocks	Deposition Temperature	Flow Rate	Pulsing Time	Purging Time	GPC (Å/cycle)	Fitted GPC (Å/cycle)	Residual (Å/cycle)
*ad*	26	1	1	1	−1	−1	1	0.96	1.02	−0.06
*ad*	10	2	1	1	−1	−1	1	0.97	1.02	−0.05
*acd*	30	3	1	1	−1	1	1	1.16	1.17	0.00
*bcd*	15	4	1	−1	1	1	1	1.27	1.31	−0.04
*bc*	23	5	1	−1	1	1	−1	1.32	1.22	0.10
*c*	5	6	1	−1	−1	1	−1	1.08	1.17	−0.09
*bd*	11	7	1	−1	1	−1	1	1.22	1.22	0.00
*ab*	20	8	1	1	1	−1	−1	1.04	1.07	−0.03
*abc*	8	9	1	1	1	1	−1	1.10	1.12	−0.02
*abc*	24	10	1	1	1	1	−1	1.10	1.12	−0.02
*abcd*	32	11	1	1	1	1	1	1.02	1.05	−0.02
*bd*	27	12	1	−1	1	−1	1	1.19	1.22	−0.03
*abd*	12	13	1	1	1	−1	1	0.95	0.86	0.09
*bcd*	31	14	1	−1	1	1	1	1.26	1.31	−0.04
*d*	25	15	1	−1	−1	−1	1	1.26	1.29	−0.03
*ac*	22	16	1	1	−1	1	−1	1.22	1.16	0.06
*c*	21	17	1	−1	−1	1	−1	1.00	1.17	−0.18
*d*	9	18	1	−1	−1	−1	1	1.28	1.29	−0.01
*b*	3	19	1	−1	1	−1	−1	1.23	1.27	−0.04
*ac*	6	20	1	1	−1	1	−1	1.22	1.16	0.06
*bc*	7	21	1	−1	1	1	−1	1.32	1.22	0.09
*(1)*	1	22	1	−1	−1	−1	−1	1.34	1.26	0.08
*cd*	13	23	1	−1	−1	1	1	1.40	1.34	0.07
*cd*	29	24	1	−1	−1	1	1	1.42	1.34	0.08
*a*	18	25	1	1	−1	−1	−1	1.15	1.15	0.00
*acd*	14	26	1	1	−1	1	1	1.17	1.17	0.00
*(1)*	17	27	1	−1	−1	−1	−1	1.34	1.26	0.08
*abcd*	16	28	1	1	1	1	1	1.00	1.05	−0.04
*a*	2	29	1	1	−1	−1	−1	1.14	1.15	−0.01
*b*	19	30	1	−1	1	−1	−1	1.22	1.27	−0.05
*abd*	28	31	1	1	1	−1	1	0.95	0.86	0.09
*ab*	4	32	1	1	1	−1	−1	1.04	1.07	−0.03

**Table 6:** Coefficients of the regression model with up to two-way interactions.

Term	Effect	Coefficient
Constant		1.1668
Deposition Temperature	−0.1858	−0.0929
Flow Rate	−0.0556	−0.0278
Pulsing Time	0.0485	0.0243
Purging Time	−0.0244	−0.0122
Deposition Temperature × Flow Rate	−0.0444	−0.0222
Deposition Temperature × Pulsing Time	0.0499	0.0250
Deposition Temperature × Purging Time	−0.0797	−0.0398
Flow Rate × Pulsing Time	0.0200	0.0100
Flow Rate × Purging Time	−0.0398	−0.0199
Pulsing Time × Purging Time	0.0681	0.0340

**Table 7:** ANOVA of the regression model with up to two-way interactions. Note that bold items are statistically significant effects.

Source	DF	SS	MS	*F*-Value	*p*-Value
Model	10	0.463938	0.046394	7.73	0.000
Linear	4	0.324541	0.081135	13.52	0.000
**Deposition Temperature**	**1**	**0.276187**	**0.276187**	**46.03**	**0.000**
Flow Rate	1	0.024741	0.024741	4.12	0.055
Pulsing Time	1	0.018841	0.018841	3.14	0.091
Purging Time	1	0.004772	0.004772	0.80	0.383
Two-Way Interactions	6	0.139397	0.023233	3.87	0.009
Deposition Temperature × Flow Rate	1	0.015741	0.015741	2.62	0.120
Deposition Temperature × Pulsing Time	1	0.019931	0.019931	3.32	0.083
**Deposition Temperature** × **Purging Time**	**1**	**0.050763**	**0.050763**	**8.46**	**0.008**
Flow Rate × Pulsing Time	1	0.003207	0.003207	0.53	0.473
Flow Rate × Purging Time	1	0.012673	0.012673	2.11	0.161
**Pulsing Time** × **Purging Time**	**1**	**0.037082**	**0.037082**	**6.18**	**0.021**
Error	21	0.125998	0.006000		
Lack-of-Fit	5	0.121292	0.024258	82.48	0.000
Pure Error	16	0.004706	0.000294		
Total	31	0.589936			

## Data Availability

The original contributions presented in this study are included in the article. Further inquiries can be directed to the corresponding author.

## Abbreviations

The following abbreviations are used in this manuscript:

ALD	Atomic Layer Deposition
DOE	Design of Experiments
ANOVA	Analysis of Variance
